# Epidemiological and clinical characteristics of severe hand-foot-and-mouth disease (HFMD) among children: a 6-year population-based study

**DOI:** 10.1186/s12889-020-08961-6

**Published:** 2020-05-27

**Authors:** Yanhao Wang, Han Zhao, Rong Ou, Hua Zhu, Lidan Gan, Zihuan Zeng, Ruizhu Yuan, Huan Yu, Mengliang Ye

**Affiliations:** 1 Department of Epidemiology and Health Statistics, School of Public Health and Management, Chongqing Medical University, Chongqing, 400016 China; 2 College of Stomatology, Chongqing Medical University, Chongqing, 401147 China; 3 Chongqing Municipal Center for Disease Control and Prevention, Chongqing, No. 8, Changjiang 2nd Road, Chongqing, 400042 Yuzhong District China; 4 Library, Chongqing Medical University, Chongqing, 400016 China; 5 Pediatric College, Chongqing Medical University, Chongqing, 400014 China; 6 College of Nursing, Chongqing Medical University, Chongqing, 400016 China

**Keywords:** Epidemiological characteristics, Risk factors, Hand-foot-and-mouth disease (HFMD), Children

## Abstract

**Background:**

Hand-foot-and-mouth disease (HFMD) is considered to be self-limited, however, severe HFMD is a deadly threat for children worldwide, therefore, it is essential to define the clinical and epidemiologic characteristics of children with severe HFMD and identify the risk factors of death.

**Methods:**

Between 2013 and 2018, children who diagnosed with severe HFMD from Chongqing, China were enrolled in this population-based study. A total of 459 severe HFMD children cases were identified during the study period, including 415 survivors and 44 fatal cases. Demographic, geographical, epidemiological and clinical data of the cases were acquired and analyzed.

**Results:**

Risk factors of the death because of severe HFMD children included female, aged 1 ~ 3 years, enterovirus 71 infection, falling ill in winter, more than one children in home, being taken care of by grandparents, the caregivers’ education not more than 9 years, having fever more than 3 days, consciousness disorders, general weakness, vomiting, general weakness, abnormal pupillary light reflex, repeated cough, tachypnea, moist rales, white frothy sputum, pink frothy sputum, and cyanosis on lips or the whole body, tachycardia, arrhythmia, cold limbs, pale complexion, weakened pulse. (all *p* < 0.05). Spatial-temporal analysis detected high-value clusters, the most likely cluster located at rural countries in the northern parts of Chongqing, from January, 2015 to July, 2017. (*p* < 0.01). Besides, some urban districts were also found high incidence of severe HFMD cases according to the incidence maps.

**Conclusions:**

The detection of clinical risk factors and the temporal, spatial and socio-demographic distribution epidemiological characteristics of severe HFMD contribute to the timely diagnosis and intervention, the results of this study can be the reference of further clinical and public health practice.

## Background

As a common infectious disease among children, hand-foot-mouth disease (HFMD) has aroused wide attention all over the world [1–5]. HFMD is mainly caused by a group of enterovirus, mainly by enterovirus 71(EV71) and coxsackie virus A16 (Cox A16) [4, 6–8], it is characterized by fever, oral ulcers, and skin eruptions on hands, feet, buttocks [9–11], and transmission occurs through the direct contact with saliva, feces, vesicular fluid, or respiratory droplets of the infected individual, and indirectly by contaminated articles [12, 13].

Generally, the state of HFMD is mild and self-limited. However, many countries or regions have experienced pandemics of severe or fatal HFMD cases in recent years, especially Asia-Pacific countries [14–18]. For China, HFMD is also a major public health problem, and the study of Xing W, et al. showed that about 500 ~ 900 people in China died because of the severe HFMD every year, mainly in children [1], and the mortality rate among children with severe HFMD is also high worldwide [19–22]. Severe HFMD poses a great threat to one’s health and life, especially the child, as well as bringing psychological and financial burden burdens for families, therefore, the research and investigation of severe HFMD are important.

Previous studies about HFMD were mainly focused on the features of mild HFMD, therefore, the incidence of HFMD worldwide has been controlled to a certain degree [23–25]. However, to our knowledge, relevant researches about the clinical or epidemiological characteristics of severe HFMD patients or fatal cases was few, which may impede the further reduction in case fatality. Since Chongqing is one of the districts in China affected by the outbreaks of severe HFMD [1, 24] and studies have shown the incidence of HFMD in Chongqing was higher than the national incidence, as well as that in many countries or regions [24], the present study aimed to investigate the clinical and epidemiological characteristics of severe HFMD and identify risk factors of severe HFMD in our population by taking Chongqing as an example, which may provide theoretical support for further prevention, diagnosis and treatment of severe HFMD.

## Methods

### Clinical definition

A clinical case of HFMD was defined as a patient with maculopapular or vesicular rash on hands, feet, mouth, or buttocks, with or without fever, according to the guidelines of the National Health Commission of the People’s Republic of China [26]. Children (aged 0–17 years) were diagnosed with severe HFMD if they developed at least one of the following: cardiopulmonary collapse, pulmonary hemorrhage, pulmonary edema, encephalitis, aseptic meningitis, acute flaccid paralysis, myocarditis, or death [27–30].

### Data collection

On 2 May2008, HFMD became a notifiable disease as a Class C (third level of severity and importance of public health) infectious disease in mainland China. Since then, all HFMD cases diagnosed in hospitals were reported to “National Disease Reporting Information System” (NDRIS) within 24 h after diagnoses, including hospitalized and ambulatory cases, not only referred cases, but also self-presenting cases, according to the requirement of the law of the People’s Republic of China on Prevention and Treatment of Infectious Diseases [23]. Therefore, we analyzed the clinical and epidemiological data of severe HFMD children patients from 1 January 2013 to 31 December 2018 in Chongqing.

### Specimen collection and laboratory testing

Appropriate clinical specimens from the severe HFMD cases, including fecal sample, rectal swab, throat swab, vesicular fluid, and/or cerebrospinal fluid, were collected. Specimens were placed in 3 mL of sterile viral transport medium and sent to biosafety level two facilities for RT-PCR test within 48 h of collection, using commercially available pan-enterovirus, EV71, and Cox A16 diagnostic kits, according to standardized protocols disseminated by the National Center for Disease Control and Prevention (CDC) [23].

### Statistical analysis

**Fig. 1:**
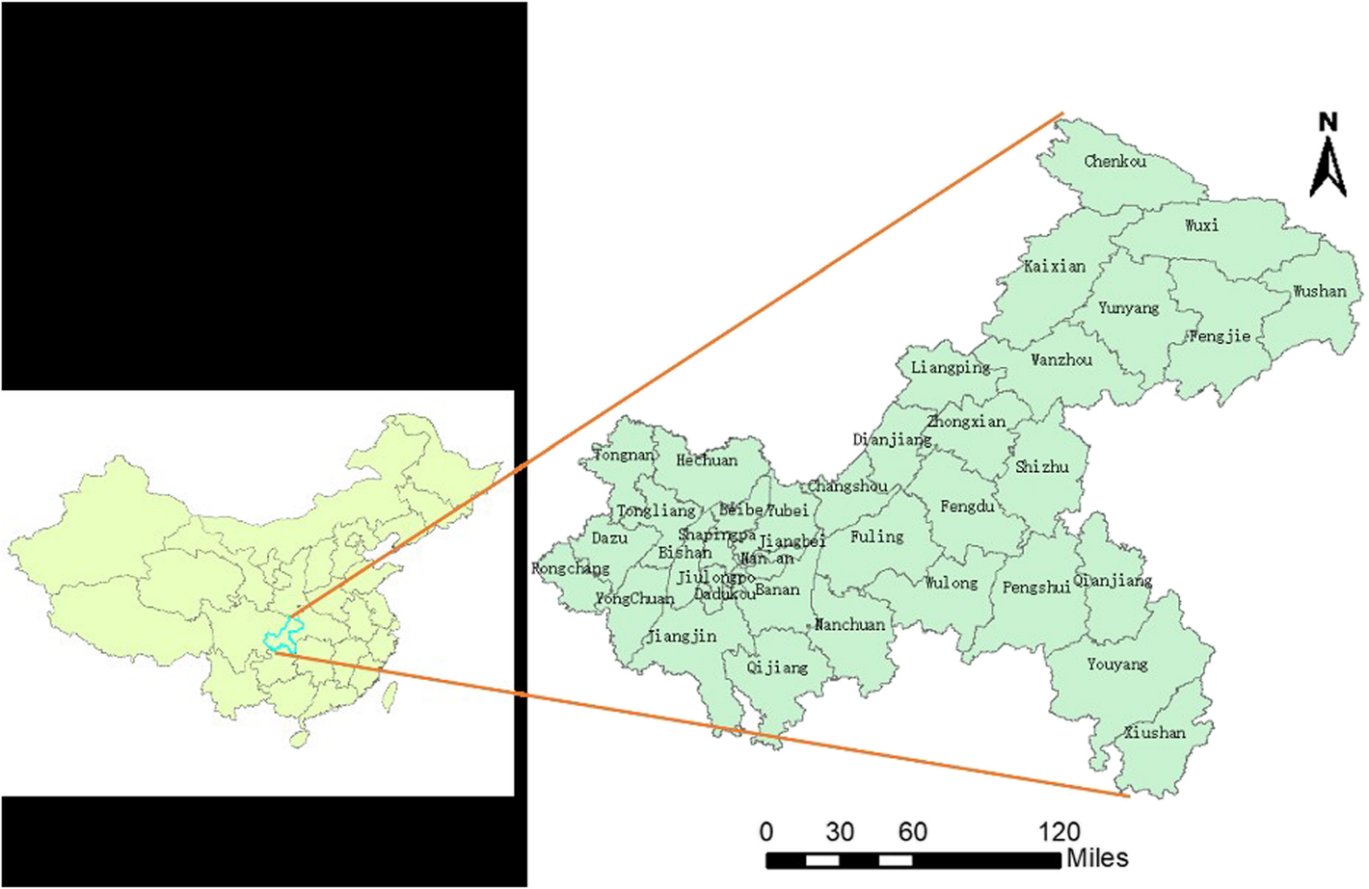
The distribution of districts and counties in Chongqing, China and its location. The study site is Chongqing, which is located in southwest of China, lying between latitude 28°10′-32°13′ N and longitude 105°11′-110°11′ E. The maps depicted in Fig. 1 were taken from National Geomatics Center of China. (http://www.ngcc.cn/ngcc/)

The study site is Chongqing, which is located in southwest of China, lying between latitude 28°10′-32°13′ N and longitude 105°11′-110°11′ E. (Fig. 1). The incidence rates of severe HFMD were computed at district level and colored with different colors in the incidence map using the software ArcGIS10.2 (ESRI, Redlands, CA, USA) to show the spatial distribution of severe HFMD.

The incidence data of severe HFMD from 2013 to 2018 were used for retrospective spatiotemporal analysis. The size of the maximum spatial cluster was defined as a circle with a radius of 92.87 km. The method is based on the geographic coordinates to establish a two-dimensional window of the cylinder. The base circle of the cylinder represents the geographical range and the height represents the length of time. The position and height of the cylinder are dynamically changing. Log likelihood ratio (LLR) was used as the index to measure the abnormal degree of severe HFMD in the scanning window and the Monte Carlo method was used to test LLR for *p* values. When *P* < 0.05, we thought that the statistical index of the scanning window compared with outside the window, the relative risk (relative risk, RR) was statistically significant. Parameters were set as: no geographical region overlap, 20% of population at risk, time length was limited to 1 month, and the number of Monte Carlo replication was 999. The areas with the largest LLR values obtained from the scan are called the most significant, and the remaining statistically significant aggregation areas are called secondary potential clusters.

The statistical analyses of clinical data were performed between the fatal cases and survivors using the SPSS software version 19.0 (IBM Corporation, Armonk, NY). We used the chi-square test for analyzing categorical data. General characteristics, family characteristics, symptoms, complications were compared in the two groups, and we performed conditional univariable logistic regression to find predictors that were discriminatory for the death because of severe HFMD [19, 31]. The level of statistical significance for all analyses was *P* < 0.05.

### Ethics statement

All the study procedures were reviewed and approved by the Ethics Committee of Chongqing Center for Disease Control and Prevention. All individual identifying information such as name, address, and telephone number, etc. was anonymized and de-identified prior to analysis. Informed consents were obtained from the individuals or their guardian before the information and the specimens were collected.

## Results

### General characteristics

A total of 459 severe HFMD cases among children were identified during the study period (2013 ~ 2018), including 415 survivors and 44 fatal cases. The mean age of the patients was 2.15 years (range from 1 months to 16 years), and the ratio of male to female was 1.8:1. Table 1 outlines the general characteristics of all cases including gender, age, the hosting classification of children, identification of enterovirus and the onset season.

**Table 1:** Demographic characteristics of severe HFMD children patients

	Fatal cases N2 = 44	Survivors N1 = 415	Total ***N*** = 459	OR (95%CI)
**Gender**				
**Male**	22 (50.0)	273 (65.8)	295 (64.3)	0.52 (0.28–0.97)*
**Female**	22 (50.0)	142 (34.2)	164 (35.7)	Referent
**Age**				
≤ **1**	4 (9.1)	47 (11.3)	51 (11.1)	0.78 (0.27–2.29)
**1~**	36 (81.8)	259 (62.4)	295 (64.3)	2.71 (1.23–5.98)**
**3~**	3 (6.8)	97 (23.4)	100 (21.8)	0.24 (0.07–0.79)*
**> 5**	1 (2.3)	12 (2.9)	13 (2.8)	Referent
**Hosting Classification**				
**Scatter children**^**a**^	36 (81.8)	333 (80.2)	369 (80.4)	1.11 (0.50–2.47)
**Childcare**^**b**^	8 (18.2)	75 (18.1)	83 (18.1)	1.01 (0.45–2.26)
**In school**^**c**^	0 (0.0)	3 (0.7)	3 (0.7)	–
**Others**	0 (0.0)	4 (1.0)	4 (0.9)	–
**Identification of enterovirus**				
EV71	30 (68.2)	216 (52.0)	246 (53.6)	1.97 (1.02–3.83)*
Cox A16	0 (0.0)	14 (3.4)	14 (3.1)	–
Other enterovirus	4 (9.1)	66 (15.9)	70 (15.3)	0.53 (0.18–1.53)
Negative	10 (22.7)	119 (28.7)	129 (28.1)	0.73 (0.35–1.53)
**Onset season**				
**Winter**	9 (20.5)	34 (8.2)	43 (9.4)	2.88 (1.28–6.49)*
**Autumn**	3 (6.8)	37 (8.9)	40 (8.7)	0.75 (0.22–2.53)
**Summer**	12 (27.3)	173 (41.7)	185 (40.3)	0.53 (0.26–1.05)
**Spring**	20 (45.5)	171 (37.3)	191 (41.6)	1.19 (0.64–2.22)
**Family members, > 3**	28 (63.6)	299 (72.0)	327 (71.2)	0.68 (0.35–1.30)
**Children in home, > 1**	37 (84.1)	275 (66.3)	312 (68.0)	2.69 (1.17–6.19)*
**Caregivers**				
**Parents**	21 (47.7)	285 (68.7)	306 (66.7)	0.42 (0.22–0.78)*
**Grandparents**	23 (52.3)	130 (31.3)	153 (33.3)	Referent
**Caregivers’ education**				
**> 9 y**	5 (11.4)	172 (41.4)	177 (38.6)	0.18 (0.07–0.47)**
**0–9 y**	39 (88.6)	243 (58.6)	282 (61.5)	Referent
**Neighborhood children also suffer from HFMD disease**	1 (2.3)	50 (12.0)	51 (11.1)	0.14 (0.02–1.01)*

**Note:***OR* Odds ratio, *CI* Confidence interval. *P < 0.05; ** *P* < 0.01

Overall, female children (odds ratio (OR) = 1.92, 95% confidence interval (CI) = 1.03–3.59, *P* < 0.05) and the children aged 1 to 3(OR = 2.71, 95%CI =1.23–5.98, *P* < 0.01) are easier to die of severe HFMD. Enterovirus 71 infection (OR = 1.97, 95%CI = 1.02–3.83, *P* < 0.05) and falling ill in winter (OR = 2.88, 95%CI = 1.28–6.49, *P* < 0.05) are the risk factors for death. The influence of hosting classification on mortality was not statistically significant. (Table 1.)

The poor prognosis (death) may associated with more than one children in the children patient’s home (OR = 2.69, 95%CI =1.17–6.19, *P* < 0.05), being taken care of by grandparents (OR = 2.40, 95%CI = 1.28–4.49, *P* < 0.01) and the caregivers’ education not more than 9 years (OR = 5.52, 95%CI = 2.13–14.29, *P* < 0.01). Besides, the children patients whose neighborhood also suffer from HFMD disease, may have a better chance of survival (OR = 0.14, 95%CI = 0.02–1.01, *P* < 0.01). (Table 1.)

### Symptoms, and signs

Table 2 shows that the children with severe HFMD who having fever more than 3 days (OR = 2.27, 95%CI = 1.14–4.54, *P* < 0.05) are easier to die. However, the children patients who had rash more than 3 days (OR = 0.50, 95%CI = 0.26–0.96, *P* < 0.05), or who were found herpes in the oral cavity (OR = 0.43, 95%CI = 0.23–0.80, *P* < 0.05) or on cheek (OR = 0.22, 95%CI = 0.09–0.58, *P* < 0.05), had a better prognosis. (Table 2.)

**Table 2:** The comparison on general symptoms and nervous, respiratory and circulatory complications between survivors and fatal cases

	Fatal cases N2 = 44	Survivors N1 = 415	Total N = 459	OR(95%CI)
Fever	42 (95.5)	396 (95.4)	438 (95.4)	1.01 (0.23–4.48)
Hyperpyrexia(T > 39 °C)	9 (20.5)	102 (24.6)	111 (24.0)	0.79 (0.37–1.70)
Fever≥3 days	32 (72.7)	224 (54.0)	286 (62.3)	2.27 (1.14–4.54)*
Rash	36 (81.8)	373 (90.0)	409 (89.1)	0.51 (0.22–1.16)
Rash≥3 days	16 (36.4)	221 (53.3)	237 (51.6)	0.50 (0.26–0.96)*
Herpes in the oral cavity	22 (50.0)	291 (70.1)	313 (68.2)	0.43 (0.23–0.80)*
Herpes on cheek	5 (11.4)	152 (36.6)	157 (34.2)	0.22 (0.09–0.58)*
Herpes on fauces	14 (31.8)	126 (30.4)	140 (30.5)	1.07 (0.55–2.09)
**Nervous complications**				
Consciousness Disorders	22 (50.0)	45	67	8.22 (4.22–16.02)**
Lethargy	33 (75.0)	307	340	1.06 (0.52–2.16)
Abnormal pupillary light reflex	11 (25.0)	22	33	5.96 (2.66–13.34)**
Panic	26	270	296	0.78 (0.41–1.46)
Vomiting	23	105	128	3.23 (1.72–6.08)**
Convulsion	13	90	51	1.51 (0.76–3.01)
General weakness	10	50	60	2.51 (1.22–5.16)*
Dysphoria	9	64	73	1.41 (0.65–3.08)
Shake of hand or foot	19	212	231	0.73 (0.39–1.36)
**Respiratory complications**				
Persistent cough	16	90	106	2.63 (1.07–3.98)*
Polypnea	9	55	64	1.68 (0.77–3.69)
Tachypnea	14	30	42	5.99 (2.87–12.49)**
Moist rales	19	40	59	7.13 (3.61–14.06)**
White frothy sputum	4	10	14	4.05 (1.22–13.50)*
Pink frothy sputum	19	14	33	21.77 (9.78–48.44)**
Cyanosis (lips)	34	87	121	12.82 (6.09–26.96)**
Cyanosis (the whole body)	13	28	41	5.80 (2.73–13.30)**
**Circulatory complications**				
Tachycardia	25 (56.8)	158 (38.1)	183	2.14 (1.14–4.01)*
Arrhythmia	13 (29.5)	25 (6.0)	38	6.54 (3.05–14.04)**
Cold limbs	17 (38.6)	42 (10.1)	59	5.59 (2.82–11.10)**
Pale complexion	18 (40.9)	48 (11.6)	66	5.29 (2.70–10.37)**
Weakened pulse	14 (31.8)	23 (5.5)	37	7.95 (3.72–14.03)**

**Note:** OR = odds ratio; CI = confidence interval. *P < 0.05, ** *P* < 0.01

### Clinical complications

The fatal cases had a higher incidence of consciousness disorders (OR = 8.22, 95%CI = 4.22–16.02, *P* < 0.01), abnormal pupillary light reflex (OR = 5.96, 95%CI = 2.66–13.34, *P* < 0.01), vomiting (OR = 3.23, 95%CI = 1.72–6.08, *P* < 0.01), and general weakness (OR = 2.51, 95%CI = 1.22–5.16, *P* < 0.05), compared with the survivors. Lethargy, convulsion and dysphoria were not significantly associated with a fatal course. (Table 2.)

The fatal cases had a higher incidence of some respiratory and circulatory complications, such as persistent cough (OR = 2.63, 95%CI = 1.07–3.98, *P* < 0.05), tachypnea (OR = 2.40, 95%CI = 1.28–4.49, *P* < 0.01), Moist rales (OR = 7.13, 95%CI = 3.61–14.06, *P* < 0.01), White frothy sputum (OR = 4.05, 95%CI = 1.22–13.50, *P* < 0.05), pink frothy sputum (OR = 21.77, 95%CI = 9.78–48.44, *P* < 0.01), lips cyanosis (OR = 12.82, 95%CI = 6.09–29.96, *P* < 0.01), the whole body cyanosis (OR = 5.80, 95%CI = 2.73–13.30, *P* < 0.01), increased heart rate (OR = 2.14, 95%CI = 1.14–4.01, *P* < 0.05), arrhythmia (OR = 6.54, 95%CI = 3.05–14.04, *P* < 0.01), cold limbs (OR = 5.59, 95%CI = 2.82–11.10, *P* < 0.01), pale complexion (OR = 5.29, 95%CI = 2.70–10.37, *P* < 0.01) and weakened pulse (OR = 7.95, 95%CI = 3.72–14.03, *P* < 0.01). (Table 2.)

### Temporal distribution of severe HFMD

**Fig. 2:**
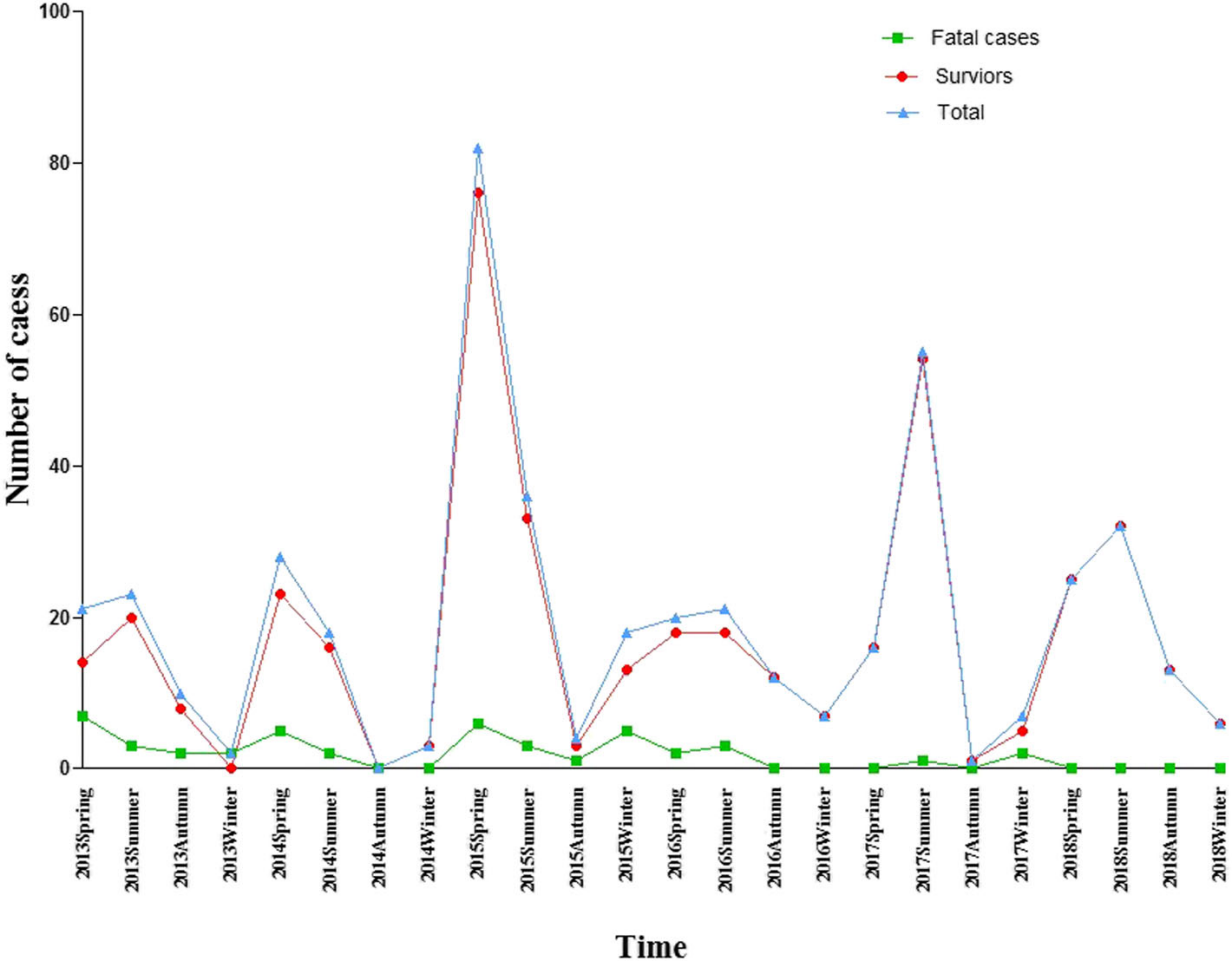
Seasonal distribution of severe HFMD children (survivors and fatal cases). Epidemic peaks of severe HFMD were commonly seen in the spring or summer, the highest number of cases reported was in the spring of 2015, and the second highest was in the summer of 2017. Fatal cases could be seen in for seasons

Figure 2 shows the temporal (seasonal) distribution of severe HFMD children from the spring of 2013 to the winter of 2018. Epidemic peaks of severe HFMD were commonly seen in the spring or summer, the highest number of cases reported was in the spring of 2015, and the second highest was in the summer of 2017. Fatal cases could be seen in for seasons. (Fig. 2.)

### Spatial distribution of severe HFMD

**Fig. 3:**
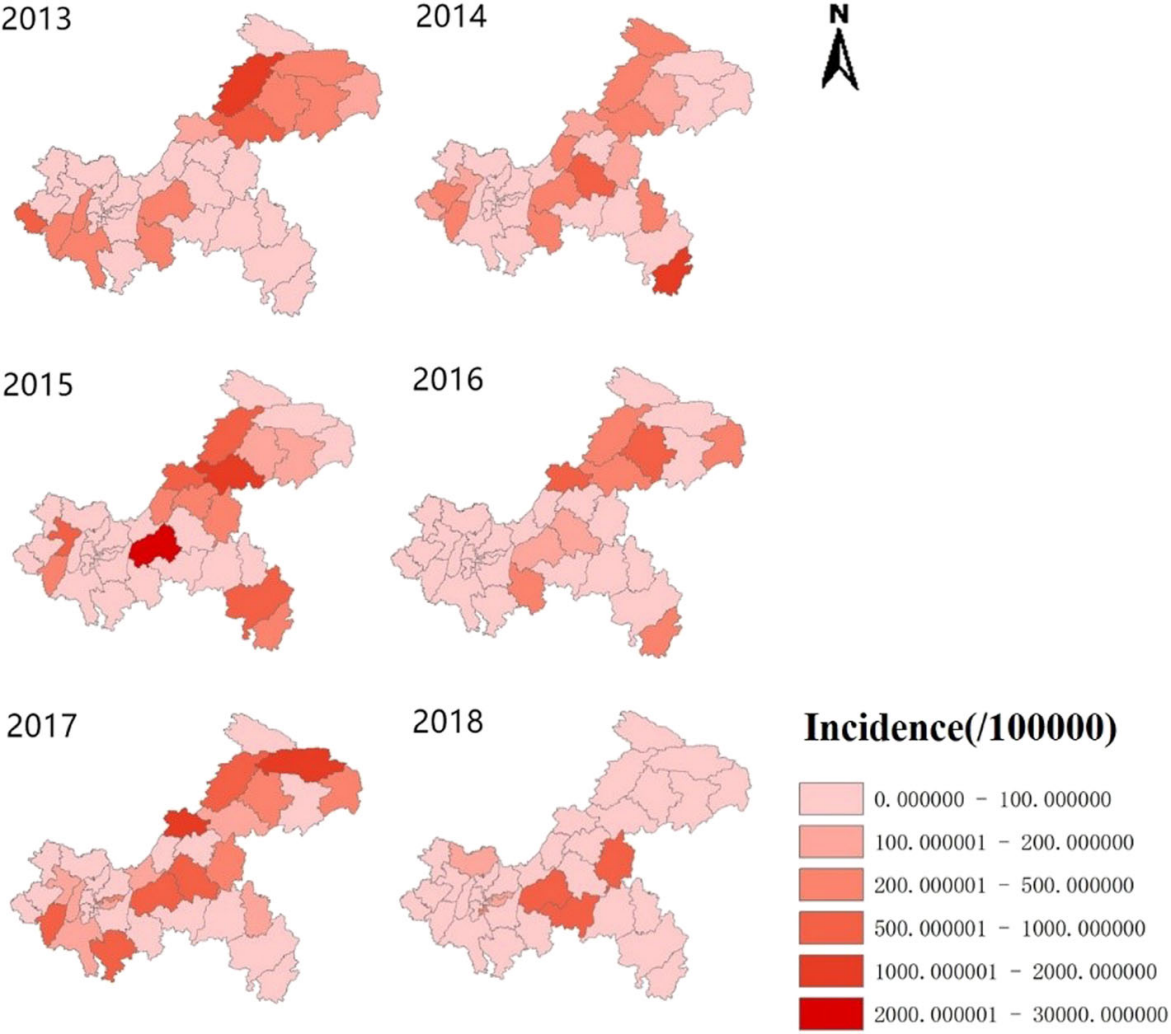
Spatial distribution of severe HFMD children (survivors and fatal cases). Overall, the thematic map of the annual incidence indicated that the northeast and middle parts of Chongqing were still the high incidence regions of HFMD, especially some counties such as Kaixian, Fuling, Wuxi, Liangping, Xiushan. Furthermore, 2013, 2015and 2017 were the relatively severe years of HFMD epidemic. The maps depicted in Fig. 3 were taken from National Geomatics Center of China. (http://www.ngcc.cn/ngcc/)

From 2013 to 2018, a total of 459 cases of severe HFMD children were discovered in Chongqing, covering 38 regions, with a total population of 2944, 9983 and an incidence rate of 0.3/100,000. Figure 3 shows the annual incidence of severe HFMD from 2013 to 2018. In the study period, the incidence of HFMD in Chongqing was randomly distributed as a whole. According to the analysis of the incidence of HFMD in Chongqing, the first three high incidence counties were Fuling, Kaixian and Wanzhou successively. (Fig. 3.)

The results showed that the higher incidence areas (dark orange) were mainly located in the northeast regions (Kaixian, Wanzhou, Wuxi, Liangping) except 2014 and 2018. In 2014, the highest incidence region was Xiushan which was on the far southeast, and higher incidence areas (light orange and yellow) varied each year and spread all over the municipality except 2018. Most of the areas with relatively high incidence were concentrated in the northeast, while the incidence in the south is relatively low. Overall, the thematic map of the annual incidence indicated that the northeast and middle parts of Chongqing were still the high incidence regions of HFMD, especially some counties such as Kaixian, Fuling, Wuxi, Liangping, Xiushan. Furthermore, 2013, 2015and 2017 were the relatively severe years of HFMD epidemic. (Fig. 3.)

### Spatial-temporal clusters

Table 3 listed the scanning results of most likely clusters for serve HFMD cases derived from the retrospective Spatial-Temporal analysis. *P* < 0.05 and the higher value of LLR, the higher risk of disease exists in the scanning window, and the window is more likely a cluster. There were 10 spatiotemporal aggregation regions with statistical significance (*P* < 0.05), including 6 from 2015 to 2017 and 4 in 2018. The most likely cluster was Kaixian, Yunyang, Wanzhou, Chengkou and Liangping from January, 2015 to July, 2017. During this period, the expected cases in this area was 30.86, but the actual number was 153, with a relative risk of 6.93, and *P* < 0.05. Secondly, the clusters centered in Fuling district from March to July in 2015, and four main urban districts (Yuzhong, Nanan, Dadukou and Jiangbei) in July 2018. (Table 3.)

**Table 3:** The scanning results of Spatial-Temporal Clusters for serve HFMD cases among children in Chongqing, 2013–2018

Cluster type	Time frame	Cluster areas	Observed cases	Expected cases	Radius/(km2)	RR	LLR	Specific districts	p
**Most likely**	2015/1–2017/7	5	153	30.86	92.87	6.93	142.123460	Kaixian, Yunyang, Wanzhou, Chengkou, Liangping	0.001*
**Secondary**	2015/3–2015/7	1	50	1.18	0	47.58	141.361975	Fuling	0.001*
**2nd Secondary**	2018.7	4	11	0.54	17.55	20.75	22.766941	Yuzhong, Nanan, Dadukou. Jiangbei	0.001*

**Note**: *LLR* Log likelihood ratio, *RR* Relative risk. * *p* < 0.01

**Fig. 4:**
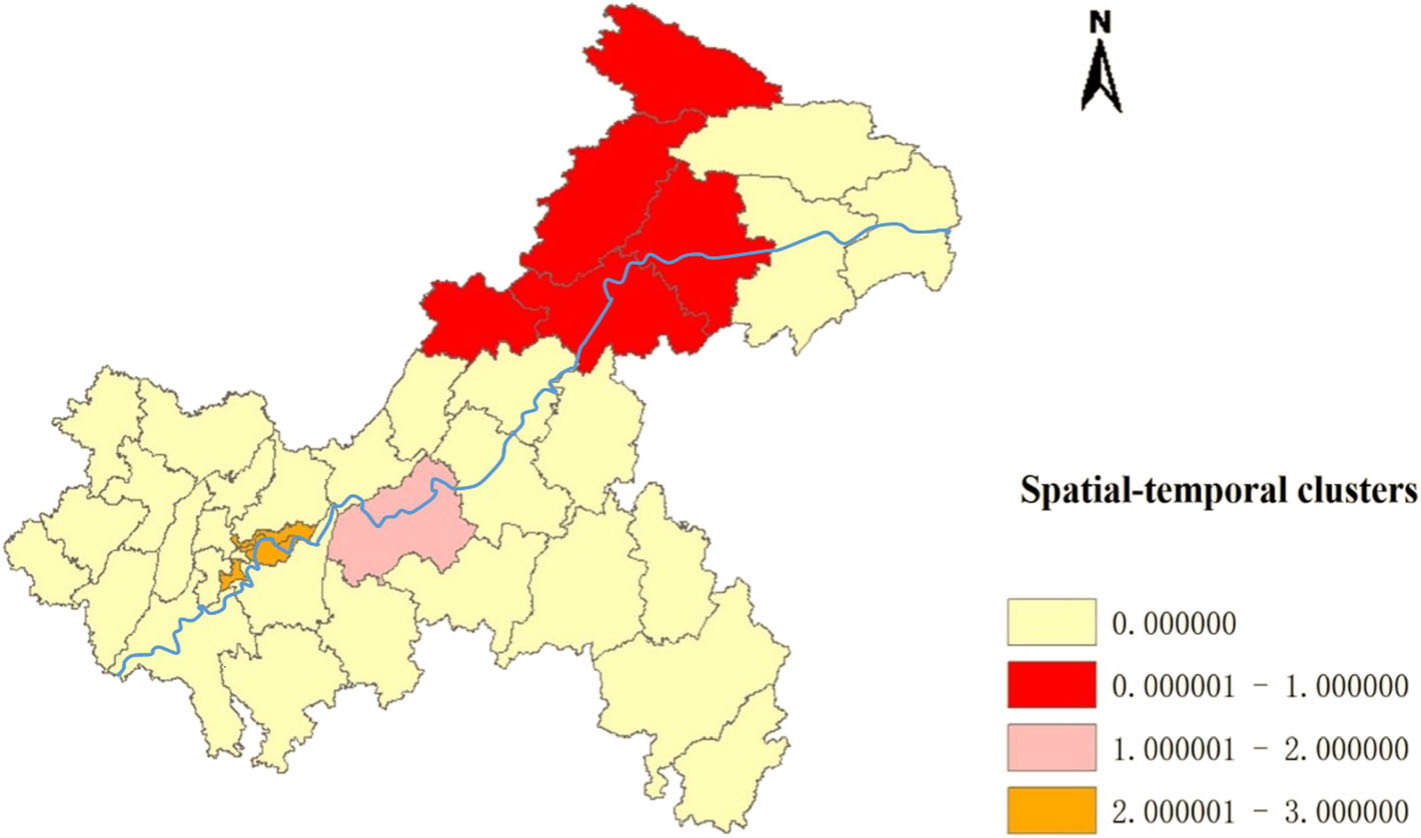
Spatial clusters of severe HFMD in Chongqing, China from 2013 to 2018. The space scan proposed by Kulldorff is integrated in SaTScan TM v9.4 (http://www.satscan.org/) and clusters are depicted on the map using the software ArcGIS10.2 (https://www.arcgis.com/features/index.html, ESRI, Inc., Redlands, CA, USA). Note: The most likely cluster was shown in red color on the map, the secondary cluster was in pink color and the 2nd secondary cluster was in orange color. The blue curve on the map represents the trunk of the Yangtze River. The map depicted in Fig. 4 were taken from National Geomatics Center of China. (http://www.ngcc.cn/ngcc/)

In Fig. 4, the most likely cluster was shown in red color on the map, the secondary cluster was in pink color and the 2nd secondary cluster was in orange color. The three clusters in our study were near the Yangtze River basin. (Fig. 4.)

## Discussion

Severe hand-foot-mouth disease progresses rapidly and may develop severe complications which could be life-threatening [32–35]. Therefore, screening children with severe HFMD for the abnormal vital signs is important in predicting impending deadly complications and allowing the timely initiation of appropriate interventions [31]. This study aims to describe the clinical and epidemiological features of severe hand-foot-and-mouth disease and identify the risk factors of death in severe HFMD children.

Among the five general characteristics in this study, aged 1 ~ 3 years, enterovirus type 71 infections and failing ill in winter, were the risk factors for the death. Similar to previous reports, in this study, most of the children patients of severe HFMD were under 3 years old, which is probably because the immune function of these children are not yet mature [36–38]. Enterovirus 71 infection is the main strain of severe and fatal HFMD all over the world [39–41]. Analogously, children with EV71 infection accounted for a relatively high proportion of fatal cases in this study, which may be related to the virulence and pathogenicity of EV71 [4]. EV71 was reported to be strongly neurotropic, it can cause damage to cerebral cortex, pons, brainstem, cerebellum and spinal cord [42]. And the injury of central nervous system can cause the abnormality of sympathetic nerve activity, causing clinical symptoms like consciousness disorders, convulsion, etc., while the blood pressure and the heart rate of the patient also change [10]. However, the onset of EV-71 infection was reported to be insidious and cause rapid progression of neurological damage, sometimes it can lead to death within the day of detecting neurological complications [36]. Therefore, the neurological abnormalities in children with severe HFMD should be paid attention to, and the development of EV71 detection methods with high sensitivity are also needed. Moreover, it is worth noting that in this study, although the incidence of severe HFMD is higher in summer or spring, the mortality rate is higher in winter, which indicates that attention should be paid to the timely detection and treatment of HFMD in winter.

Among the five family characteristics, more than one children in home, being taken care of by grandparents and the caregivers’ education not more than 9 years, were the risk factors for the death of severe HFMD, while neighborhood children having HFMD disease, being taken care of by parents and caregivers’ education more than 9 years were the protective factors of the poor prognosis (death). These results indicate that the timely detection of severe HFMD is important and family caregivers need to be educated about hand-foot-mouth disease and related symptoms.

In the analysis of general symptoms or signs of HFMD, having fever more than 3 days is the risk factor of poor prognosis, while having rash more than 3 days, having herpes in the oral cavity or on cheek may be the protective factors, the reason is probably because people are more likely to initially diagnose HFMD by finding the rash. In the analysis of neurological complications, consciousness disorders, vomiting, abnormal pupillary light reflex, general weakness were associated with the death because of severe HFMD. When focused on respiratory complications, seven risk factors were identified to be the factors associated with the poor prognosis in this study, namely the presence of repeated cough, tachypnea, moist rales, white frothy sputum, pink frothy sputum, and cyanosis on lips or the whole body. In the analysis of circulatory complications, tachycardia, arrhythmia, cold limbs, pale complexion, weakened pulse were reported as predictive factors for a fatal course of severe HFMD.

Researches mainly attributed the death of severe HFMD to refractory heart failure and neurogenic pulmonary edema (NPE) [43–45]. At present, the mechanism of NPE is thought to be caused by specific injury in brainstem or medulla oblongata and inflammatory response [32, 46, 47]. Central nervous system lesions and inflammatory response can increase the intracranial pressure, which causes dysfunction of the optic hypothalamus and medullary solitary tract nucleus and complications such as vomiting, consciousness disorders, Convulsion, excessive sympathetic excitation, etc. occur. Then the level of serum catecholamine (epinephrine, norepinephrine, etc.) was significantly increased, so systemic vasoconstriction and hemodynamic changes sharply, systemic circulation resistance and arterial blood pressure increase sharply, and left ventricular ejection will reduce, then large amounts of blood flow from the systemic circulation will enter into the pulmonary circulation, the effective filtration pressure of pulmonary capillary will increase rapidly, and a large amount of fluid retention in the lung tissue clearance, causing severe pulmonary edema. Blood flow impact may induced vascular endothelial cell damage, vasoactive substances like histamine and bradykinin are released in large quantities, then vascular permeability increased and the plasma extravasation is massive, which intensifying the degree of pulmonary edema. After the occurrence of NPE, the ratio imbalance of receptors alpha and beta in lung tissue occurs under the excitement of sympathetic nervous, inducing the increase of the vascular permeability, as a result, inflammatory mediators such as IL-10, IL-13, IFN-γ will cause the increase of alveolar exudation, which further aggravates pulmonary edema and heart failure [48, 49]. Through the analysis of NPE mechanism, combined with the results of this study, the neurological complications might be the timely predictive factors of poor prognosis, especially consciousness disorders, general weakness, abnormal pupillary light reflex, and some respiratory and circulatory complications are thought to be the warning signal for severe HFMD children, such as white frothy sputum, pink frothy sputum, tachypnea, cyanosis on lips or the whole body; moist rales, tachycardia,arrhythmia, cold limbs, pale complexion, and weakened pulse.

Spatial aggregation of disease means that the risk of disease is significantly higher in some regions than in others. Spatial-Temporal scanning included population variables, that’s is to say, corrected the non-uniform population density in different places, and detected the clustering of cases in both time and space dimensions by moving window method. Through analyzing the severe HFMD cases from 2013 to 2018 in Chongqing, by using space-time scan, found that the clusters were mainly concentrated in Kaixian in 2013, Xiushan in 2014, Fuling and Wanzhou in 2015, and in 2017, mainly concentrated in the Wuxi and Liangping, and it is interesting to note that these areas are mainly concentrated near the Yangtze River basin, which indicates that the prevalence of severe HFMD may be associated with the flow of population, water pollution or other factors related to the river. From the perspective of overall incidence, the results showed that, the incidence of severe HFMD was relatively high in 2013, 2015 and 2017, and significantly decreased in 2014, 2016 and 2018, which may be due to the medical and economic conditions in different regions, showing a cyclical change of decrease and increase, and the prevention and control situation is severe.

As an exploratory analysis, the spatial-temporal scanning method objectively demonstrated the spatial and temporal regularity of the severe HFMD, and well evaluated the abnormal increase of HFMD in different time and space. Through the study, it was found that the incidence of serve HFMD in Chongqing from 2013 to 2018 was not randomly distributed, and there was obvious spatial and temporal aggregation. The spatial-temporal scanning analysis method makes up for the deficiency of the simple epidemiological morbidity comparison and avoids the artificial high incidence of infectious diseases. The judgment of the incidence area is more convincing than the conventional analysis. Meanwhile, combined with the geographic information system, the incidence aggregation area is more intuitive and comprehensive, providing a scientific reference for the development of targeted prevention and control measures in the future.

In conclusion, the correct and timely diagnosis of HFMD, the timely detection of severe cases, as well as timely intervention, close monitoring, and symptomatic treatment are the keys to avoid or slow down the further development of severe cases and reduce the mortality rate of severe HFMD. Besides, health education should be carried out before the high incidence period of HFMD and preventive or protective measures should be taken for children according to the temporal, spatial and socio-demographic epidemiological distribution characteristics of HFMD. Highly sensitive and rapid methods to detect the enterovirus are also needed to be developed [50, 51]. The results of this study can be the reference of further clinical and public health practice.

## Conclusions

In this study, several clinical risk factors and the temporal, spatial and socio-demographic distribution epidemiological characteristics of severe HFMD contribute to the timely diagnosis and intervention of severe HFMD. Public health or medical staff should take specific measures measures for the children according to the clinical and epidemiological characteristics of severe HFMD, the results of the present study can be the reference of further clinical and public health practice or studies.

## Data Availability

The datasets used and/or analyzed in the present study are available from the corresponding author on reasonable request.

## Abbreviations

HFMD: Hand-foot-and-mouth disease
NDRIS: National Disease Reporting Information System
EV71: enterovirus 71
Cox A16: Coxsackie virus A16
OR: Odds ratio
CI: Confidence interval
LLR: Log likelihood ratio
RR: Relative risk
CDC: Center for Disease Control and Prevention

## References

[1] Xing W, Liao Q, Viboud C (2014). Hand, foot, and mouth disease in China, 2008–12: an epidemiological study. Lancet Infect Dis.

[2] Audrey M, Vié LSF, Bruno P (2016). Ambulatory pediatric surveillance of hand, foot and mouth disease as signal of an outbreak of Coxsackievirus A6 infections, France, 2014–2015, Emerging. Infect Dis.

[3] Buttery VW, Kenyon C, Grunewald S (2015). Atypical presentations of hand, foot, and mouth disease caused by Coxsackievirus A6 — Minnesota, 2014. MMWR Morb Mortal Wkly Rep.

[4] Solomon T, Lewthwaite P, Perera D (2010). Virology, epidemiology, pathogenesis, and control of enterovirus 71. Lancet Infect Dis.

[5] Sousa IP, Burlandy FM, Costa EV (2018). Enteroviruses associated with hand, foot, and mouth disease in Brazil. J Inf Secur.

[6] Patel KP, Bergelson JM (2009). Receptors identified for hand, foot and mouth virus. Nat Med.

[7] Abedi GR, Watson JT, Pham H (2015). Enterovirus and human Parechovirus surveillance - United States, 2009-2013. MMWR Morb Mortal Wkly Rep.

[8] Samanta GP (2015). A delayed hand-foot-mouth disease model with pulse vaccination strategy. Comp Appl Math.

[9] Goksugur N, Goksugur S (2010). Images in clinical medicine. Hand, foot, and mouth disease. N Engl J Med.

[10] Esposito S, Principi N (2018). Hand, foot and mouth disease: current knowledge on clinical manifestations, epidemiology, aetiology and prevention. Eur J Clin Microbiol Infect Dis.

[11] Ooi MH, Wong SC, Lewthwaite P, Cardosa MJ, Solomon T (2010). Clinical features, diagnosis, and management of enterovirus 71. Lancet Neurol.

[12] Sharma S, Samanta GP (2017). Analysis of a hand-foot-mouth disease model. Int J Biomathematics.

[13] Phyu WK, Ong KC, Wong KT (2017). Modelling person-to-person transmission in an enterovirus A71 orally infected hamster model of hand-foot-and-mouth disease and encephalomyelitis. Emerg Microbes Infect.

[14] WHO (2011). A guide to clinical management and public health response for hand foot mouth disease (HFMD).

[15] Saki T, Qiaohong L, Van BTP (2016). Hand, foot, and mouth disease in China: modeling epidemic dynamics of enterovirus serotypes and implications for vaccination. PLoS Med.

[16] Sabanathan S, Tan LV, Thwaites L (2014). Enterovirus 71 related severe hand, foot and mouth disease outbreaks in South-East Asia: current situation and ongoing challenges. J Epidemiol Community Health.

[17] Xu W, Liu CF, Yan L (2012). Distribution of enteroviruses in hospitalized children with hand, foot and mouth disease and relationship between pathogens and nervous system complications. Virol J.

[18] Li J, Pan H, Wang X (2018). Epidemiological surveillance of hand, foot and mouth disease in Shanghai in 2014–2016, prior to the introduction of the enterovirus 71 vaccine. Emerg Microbes Infect.

[19] Chen SC, Chang HL, Yan TR (2007). An eight-year study of epidemiologic features of enterovirus 71 infection in Taiwan. Am J Trop Med Hyg.

[20] Seiff A (2012). Cambodia unravels cause of mystery illness. Lancet.

[21] Mirand A, Henquell C, Archimbaud C (2012). Outbreak of hand, foot and mouth disease/herpangina associated with coxsackievirus A6 and A10 infections in 2010, France: a large citywide, prospective observational study. Clin Microbiol Infect.

[22] Fonseca MC, Sarmiento L, Resik S (2014). Coxsackievirus A6 and enterovirus 71 causing hand, foot and mouth disease in Cuba, 2011–2013. Arch Virol.

[23] Qi L, Tang W, Zhao H (2018). Epidemiological characteristics and spatial-temporal distribution of hand, foot, and mouth disease in Chongqing, China, 2009–2016. Int J Environ Res Public Health.

[24] Yu S, Zhou Z, Yang F (2014). Temporal and spatial clustering characteristics and changes of severe hand, foot, and mouth disease in mainland of China, from 2008 to 2013. Zhonghua Liu Xing Bing Xue Za Zhi.

[25] Wang J, Hu T, Sun D (2017). Epidemiological characteristics of hand, foot, and mouth disease in Shandong, China, 2009-2016. Sci Rep.

[26] Protocol of Sample Collection and Laboratory Tests for HMD Cases. Available online:http://www.chinacdc.cn/jkzt/crb/szkb/jszl_2275/200906/t20090612_24707.htm (Accessed on 12 June 2009).

[27] Ministry of Health of the People’s Republic of China. Hand, Foot and Mouth Disease Clinic Guide (revised in 2010). Available at http://www.nhfpc.gov.cn/yzygj/s3593g/201306/6d935c0f43cd4a1fb46f8f71acf8e245.shtml/. (Accessed on 23 June 2016.).

[28] Mou J, Dawes M, Li Y (2014). Severe hand, foot and mouth disease in Shenzhen, South China: what matters most?. Epidemiol Infect.

[29] Shao-Ming C, Li Q, Zhong-Hua D (2017). Spatial clustering of severe hand-foot-mouth disease cases on Hainan Island, China. Jpn J Infect Dis.

[30] He Y, Yang J, Zeng G (2014). Risk factors for critical disease and death from hand, foot and mouth disease. Pediatr Infect Dis J.

[31] Chew S-P, Chong S-L, Barbier S, Matthew A, Lee JH, Chan YH (2015). Risk factors for severe hand foot mouth disease in Singapore: a case control study. BMC Infect Dis.

[32] Chang LY, Lin TY, Hsu KH (1999). Clinical features and risk factors of pulmonary oedema after enterovirus-71-related hand, foot, and mouth disease. Lancet.

[33] Peng CK, Tai GK, Yin CC (2003). Epidemic hand, foot and mouth disease caused by human enterovirus 71, Singapore. Emerg Infect Dis.

[34] Lin YW, Chang KC, Kao CM (2009). Lymphocyte and antibody responses reduce enterovirus 71 lethality in mice by decreasing tissue viral loads. J Virol.

[35] Samanta GP (2014). Analysis of a delayed hand–foot–mouth disease epidemic model with pulse vaccination. Syst Sci Control Eng.

[36] Qiu J, Yan HP, Cheng NC (2019). The clinical and epidemiological study of children with hand, foot, and mouth disease in Hunan, China from 2013 to 2017. Sci Rep.

[37] Liu SL, Pan H, Liu P (2015). Comparative epidemiology and virology of fatal and nonfatal cases of hand, foot and mouth disease in mainland China from 2008 to 2014. Rev Med Virol.

[38] Zhi-Yong Y, Xiu-Qi C, Dan S, 等. Mortality in children with severe hand, foot and mouth disease in Guangxi, China. Indian Pediatr. 55(2):137–9. 29242411

[39] Bible JM, Pantelidis P, Chan PKS (2007). Genetic evolution of enterovirus 71: epidemiological and pathological implications. Rev Med Virol.

[40] Chang LY, King CC, Hsu KH (2002). Risk factors of enterovirus 71 infection and associated hand, foot, and mouth disease/Herpangina in children during an epidemic in Taiwan. Pediatrics.

[41] Takechi M, Fukushima W, Nakano T (2019). Nationwide survey of pediatric inpatients with hand, foot, and mouth disease, Herpangina, and associated complications during an epidemic period in Japan: estimated number of hospitalized patients and factors associated with severe cases. J Epidemiol.

[42] Ong KC, Wong KT (2015). Understanding enterovirus 71 Neuropathogenesis and its impact on other neurotropic enteroviruses. Brain Pathol.

[43] Lum LCS, Wong KT, Lam SK (1998). Fatal enterovirus 71 encephalomyelitis. J Pediatr.

[44] Chan LG, Parashar UD, Lye MS, Ong FG, Zaki SR, Alexander JP (2000). Deaths of children during an outbreak of hand, foot, and mouth disease in Sarawak, Malaysia: clinical and pathological characteristics of the disease. For the outbreak study group. Clin Infect Dis.

[45] Lin W, Su Y, Jiang M (2017). Clinical features for 89 deaths of hand, foot and mouth disease in Guangxi, China, 2014. Int J Infect Dis.

[46] Hsuh C, Jung SM, Shih SR (2000). Acute encephalomyelitris during and outbreak of entarovirus type 71 infection in Taiwan:report of an autopsy case with pathologic immunofluoresecence, and molecular studies. Mod Pathol.

[47] Fu YC, Chi CS, Chiu YT (2004). Cardiac complications of enterovirus rhombeneephalitis. Arch Dis Child.

[48] Wang SM, Lei HY, Liu CC (2012). Cytokine immunopathogenesis of enterovirus 71 brain stem encephalitis. Clin Dev Immunol.

[49] Li X-W, Ni X, Qian S-Y (2018). Chinese guidelines for the diagnosis and treatment of hand, foot and mouth disease (2018 edition). World J Pediatr.

[50] Wang Y, Zou G, Xia A, et al. Enterovirus 71 infection in children with hand, foot, and mouth disease in Shanghai, China: epidemiology, clinical feature and diagnosis. Virol J. 2015;12:83. 10.1186/s12985-015-0308-2. 10.1186/s12985-015-0308-2 PMC4464242 26036928

[51] Zhang J, Weng Z, Du H (2016). Development and evaluation of rapid point-of-care tests for detection of enterovirus 71 and Coxsackievirus A16 specific immunoglublin M antibodies. J Virol Methods.

